# Associations between health behaviour, secondary health conditions and quality of life in people with spinal cord injury

**DOI:** 10.4102/ajod.v8i0.463

**Published:** 2019-06-11

**Authors:** Mokgadi K. Mashola, Diphale J. Mothabeng

**Affiliations:** 1 Department of Physiotherapy, University of Pretoria, Pretoria, South Africa

**Keywords:** spinal cord injury, secondary health conditions, quality of life, physical activity, health behaviour, social support, social relationships

## Abstract

**Background:**

The development of secondary health conditions (SHCs) after spinal cord injury (SCI) is common and can affect an individual’s emotional well-being, and his or her health-related quality of life (QOL). Little is known about relationships between performing health-benefiting behaviours and the presence (or absence) of SHCs and QOL, particularly in South Africa.

**Objectives:**

This research study was conducted in order to determine the associations between health behaviour, SHCs and QOL in people with SCI (PWSCI).

**Method:**

This cross-sectional study included 36 PWSCI discharged from a private rehabilitation facility in Pretoria, South Africa. The PWSCI completed questionnaires pertaining to lifestyle, independence, presence of SHCs, social support and QOL. Data were analysed using descriptive and inferential statistics such as correlation tests and chi-square test of independence (*x^2^*) using the SPSS v25. Moderate, moderately high and high correlations are reported (Pearson *r* ≥ 0.4). Results were significant if *p* < 0.05.

**Results:**

Participation in health-benefiting behaviour was associated with increased QOL (*r* = 0.457, *p* < 0.01) and increased social support from family and friends (*r* = 0.425, *p* < 0.01), which was associated with increased QOL (*r* = 0.671, *p* < 0.001). Not participating in specific neuromusculoskeletal health behaviours was found to be associated with the overall presence of SHCs (*r* = -0.426, *p* < 0.01).

**Conclusions:**

Participating in health-benefiting behaviour can reduce the development of SHCs and subsequently increase QOL in PWSCI. Health professionals must focus on minimising the development of SHCs by providing specific education on good health-benefiting behaviour.

## Introduction

### Problem statement

Spinal cord injuries (SCIs) not only limit physical function and independence but also impair psychosocial and emotional function and predispose affected individuals to secondary health conditions (SHCs) (Middleton et al. 2004). People with SCI (PWSCI) are driven to act and behave the way they do by both internal and external factors, and such behaviours may either enhance or reduce their quality of life (QOL). Social support is an external factor that is crucial to meet the challenges that PWSCI face after injury; however, studies have shown that there is a social support deficit after SCI (Müller et al. 2012). Substance abuse is linked to reduced social support, being more vulnerable to depression and reduced participation in physical activity (Middleton et al. 2004). People with SCI are known to modify their engagement in health behaviour after the development of SHCs, and not before (Bloemen-Vrencken et al. 2007). The development of SHCs may require PWSCI to perform more health behaviours in order to try and avoid future SHCs.

## Background

Secondary health conditions have been associated with reduced life expectancy, poor health outcomes and increased hospital stay (Joseph & Nilsson Wikmar 2016). Pressure ulcers (PUs), respiratory complications and urinary tract infections (UTIs) are the most common SHCs globally (Kalpakjian et al. 2007) and in South Africa (Joseph & Nilsson Wikmar 2016). Secondary health conditions affect emotional well-being and reduce social activity, community participation and health-related QOL of PWSCI (Middleton et al. 2004). Health-related QOL is further diminished by reduced participation in physical activity (PA) (sedentary lifestyle). Reduced PA can be because of numerous factors, including psychological factors, poor access to exercise facilities and impaired or loss of motor function (Stevens et al. 2008). Participation in regular PA has numerous benefits such as psychological well-being (Galea 2012), as well as reduced risk of heart disease, diabetes and obesity (Martin Ginis et al. 2012); improved cardiovascular fitness; reduced levels of depression; and consequently, an improved QOL (Ellapen et al. 2017). Despite the benefits of PA, PWSCI often spend less than 2% of their time engaging in any form of leisure-time physical activity (LTPA) (Martin Ginis et al. 2008). Not engaging in any PA presents a serious health issue in PWSCI as it increases the risk of developing SHCs (Martin Ginis et al. 2008), which further reduces the QOL of PWSCI.

### Trends

One of the ways through which PWSCI can avoid SHCs and improve QOL is LTPA, which includes playing sports, exercising at the gym or taking a stroll (Martin Ginis et al. 2012). Barriers to LTPA generally include time constraints, lack of internal motivation and not knowing which exercise to perform. However, PWSCI experience added challenges, such as locating facilities with accessible exercise equipment, shower facilities after exercise and fitness professionals who are aware of the exercise needs of PWSCI (Cowan, Nash & Anderson 2013). Physical activity aims to improve or maintain some component of physical fitness and is promoted within the SCI community as a means of improving physical health and QOL (Roberton et al. 2011).

Quality of life is an individual’s perception of his or her own position in life, which is defined by his or her own culture, values, goals, expectations and concerns (WHOQOL Group 1994). The presence of SHCs is the single important predictor of QOL, followed by societal participation (Barker et al. 2009). Poor QOL may lead to readmission to hospital, further reduced social interaction and reduced mobility. Quality of life positively influences life satisfaction, perceived social support, home and community access and participation, perceptions of having control over one’s own life and satisfaction with relationships (Hill et al. 2010). Quality relationships improve mental health and provide or receive support, while maintaining active roles in significant relationships is important for families adjusting to physical limitations (Tramonti, Gerini & Stamacchia 2015). Cowan et al. (2013) found that 84% of PWSCI did not enjoy exercise, and the reasons are multi-factorial. Personal and external factors such as motivation to exercise and accessibility of training facilities are some of the factors preventing PWSCI from enjoying exercise. More importantly, the belief that exercise will worsen the condition (or fear of injury) has been found to be associated with the reduced participation in PA. The fear is not unfounded, as exercising incorrectly (especially without supervision), and beyond the prescribed intensity, was suggested to be associated with musculoskeletal pain after commencement of exercise (Curtis et al. 1999).

### Objectives and contribution to the field

There is limited research internationally and locally on SHCs following SCIs. Only one study, conducted in Cape Town, has assessed the prevalence of SHCs in patients with SCIs in South Africa (Joseph & Nilsson Wikmar 2016). We augment the existing literature, incorporating studies from Canada and Australia, by identifying SHCs most experienced by PWSCI from Tshwane district of South Africa. We assess health behaviours, and their association with the presence or absence of SHCs, thus enabling physiotherapists to develop targeted education and prevention programmes that can be implemented while PWSCI are being rehabilitated. We determine the influence of SHCs on QOL in PWSCI.

## Research methods and design

### Design

We used a quantitative approach, a cross-sectional and correlational survey design, to determine if PWSCI performed health-promoting behaviours, whether they had any SHCs and to determine their independence levels, self-perceived QOL and perceived levels of social support. The target population included all PWSCI who were discharged from a private rehabilitation centre in the City of Tshwane (CoT) metropolitan area, from January 2008 to December 2012. The rehabilitation centre’s database was perused and PWSCI with a residential address in the CoT metropolitan area and correct contact details were included in the study. Further inclusion criteria were accessibility for the researcher and informed consent. We excluded participants younger than 18 years to avoid the involvement of a third party. As a third party, parents would have to provide consent and thus feel obliged to answer the questionnaires on the minor’s behalf. We wanted to receive accurate subjective information from the PWSCI and not from a third party. We collected data at either the participant’s homes or workplaces. The study was conducted during business hours; therefore, the setting was dependent on the participant’s preference.

#### Procedure

Non-probability convenience sampling was used, and to guarantee representativeness of the population, all PWSCI residing in the CoT metropolitan area that met the inclusion criteria were eligible to partake in the study. We identified 171 eligible participants from the patient database at the rehabilitation centre. We telephoned all the potential participants to explain the study and asked permission to visit them in their homes or workplaces to explain the study in detail. During the visit, the study was personally explained to the potential participants and signed informed consent was obtained. Only 36 participants consented to participate in the study, with 135 participants not participating as they had relocated, were no more, were unreachable or not interested. Socio-demographic data such as age, gender and injury profile were collected using a socio-demographic questionnaire. The functional independence of PWSCI was collected using the Spinal Cord Independence Measure (SCIM III). The SCIM III is a 19-item questionnaire which determines the participant’s independence in the areas of self-care (feeding, grooming, bathing and dressing), respiration, sphincter management and mobility (bed and transfers as well as indoor/outdoor mobility). The SCIM III has excellent internal consistency (Cronbach’s ∝ = 0.77–0.91) (Catz & Itzkovich 2007). Higher SCIM III scores reflect higher levels of independence. To determine if participants were functioning optimally, their SCIM III scores per neurological level of injury (NLI) were compared to expected total and median SCIM III scores for people with complete SCI by Aidinoff et al. (2011). These values can also be used as minimum target values for people with incomplete SCI. The expected total (and median scores) are as follows:

C1–C4 = 4–19.5 (8–21.5)

C5–T1 = 24–52 (23–42)

T2–T6 = 53–64.2 (60.5–57.8)

T7–T12 = 60.9–69.6 (66.3–67.5)

L1–L5 = 70.5–74.1 (72–76.3)

The Leisure-time Physical Activity Questionnaire for People with Spinal Cord Injury (LTPAQ-SCI) was used to determine if PWSCI participated in any LTPA. The LTPAQ-SCI is a three-item instrument that captures different types of LTPA performed at different intensities such as mild, moderate and heavy intensity LTPA (Martin Ginis et al. 2012). The LTPAQ-SCI showed significant test–retest reliability coefficients with all intraclass correlation coefficients (ICC) > 0.60 (with total and heavy intensity LTPA showing the strongest ICCs at 0.83 and 0.93, respectively). The scores were determined by calculating the total number of minutes per activity performed at different intensities, thereby yielding the total number of minutes of LTPA performed per day (Martin Ginis et al. 2012).

Data pertaining to health behaviour were collected using the Spinal Cord Injury Lifestyle Scale (SCILS). The SCILS is a 25-item questionnaire used to determine the participant’s engagement in health-benefiting behaviour (Pruitt et al. 1998). The SCILS has five subscales, namely cardiovascular (four items), genitourinary (four items), neuromuscular (eight items), skin (six items) and psychosocial (two items). The total SCILS internal consistency was found to be excellent (Cronbach’s *∝* = 0.81) and poor to excellent for the SCILS subscales (Cronbach’s *∝* = 0.31 to 0.86). Higher total scores are intended to indicate better performance of behaviours, which in turn promote health in PWSCI.

The Spinal Cord Injury Secondary Health Condition Scale (SCI-SCS) was used to determine the presence of SHCs. The SCI-SCS is a 16-item questionnaire, which has adequate to excellent internal consistency (Cronbach’s *∝* = 0.761 to 0.861). For each SHC listed, PWSCI were expected to rate how much each SHC affected their activities and independence in the last 3 months. Higher scores indicate greater overall problems with SHCs (Kalpakjian et al. 2007).

Data pertaining to QOL and social support were collected using the World Health Organization Quality of Life Assessment (WHOQOL-BREF) and the Social Support List (SSL-12). The WHOQOL-BREF is a 26-item questionnaire that measures both objective and subjective QOL. It is grouped into four domains of QOL (physical health, psychological health, social relationships and environment) and two items that measure the overall QOL and general health (Hill et al. 2010). The WHOQOL-BREF showed high reliability with Cronbach’s *∝* = 0.74–0.87. A score is generated for each scale by summing up the scores of each item and the total score is calculated by summing the five scale scores. Sub-scale scores were also used to identify specific areas of concern. The SSL-12 is a 12-item questionnaire used to assess PWSCIs’ perceived social support from members of their primary social network (Van Leeuwen et al. 2010). The measurement properties of the SSL-12 were found to be satisfactory with significant Spearman correlations (range 0.34–0.64; *p* < 0.01). The higher the score, the more social support perceived by PWSCI (Van Leeuwen et al. 2010).

#### Analyses

Data were captured on an Excel spreadsheet and analysed with descriptive and inferential statistics, using the SPSS v25. Socio-demographic information and the abovementioned questionnaire’s scores were analysed using frequencies, percentages, means and standard deviations. Associations between variables of interest were analysed using Pearson correlation tests and expressed as ‘*r*’. Results were statistically significant if *p* < 0.05. This manuscript reports correlations in the following manner (Safrit & Wood, cited in Zhu 2012):

- No correlation: *r* = 0 – 0.19
- Low correlation: *r* = 0.2 – 0.39
- Moderate correlation: *r* = 0.4 – 0.59
- Moderately high correlation: *r* = 0.6 – 0.79
- High correlation *r* = ≥ 0.8

Portney and Watkins (2015) recommended that low correlations not be discussed as clinically important merely owing to the achievement of statistical significance (when *p* > 0.05). Zhu (2012) further supported this and highlighted the challenges with drawing conclusions merely based on the *p*-value, as the *p*-value is biased by the sample size. This study will therefore only draw conclusions on moderate, moderately high and high correlations as described above. The chi-square test of independence (*x^2^*) was used to further determine if there were significant relationships between specific SHCs and items pertaining to health behaviour and QOL (categorical variables) (Portney & Watkins 2015).

#### Potential benefits and hazards

This study provides insights into the relationships between health behaviours, SHCs and QOL after SCI. There were no hazards for the participants, as the patients were not in any physical or psychological harm nor did the study require any physical exertion.

### Ethical considerations

We obtained informed consent from the participants prior to completing the questionnaire. The questionnaire and the reasoning for the study were explained to the participants. The researcher addressed any questions or misgivings before filling in the questionnaires to ensure that participants understood the nature of the study and their role in it. This study received ethical approval from the Faculty of Health Sciences Research Ethics Committee at the University of Pretoria (approval number 21/2014).

## Results

### Demographic information, injury profile and functional independence of people with spinal cord injury

**FIGURE 1:**
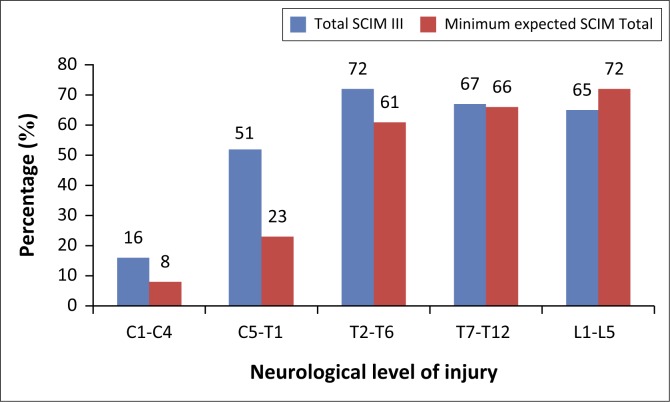
Achieved versus expected Spinal Cord Independence Measure Scale III scores of people with spinal cord injury.

Most participants were male (*n* = 24, 67%), single (*n* = 18, 50%) and between 30 and 39 years old (*n* = 10, 28%). Fewer participants were employed after SCI (*n* = 22, 61%) compared to those employed before SCI (*n* = 27, 75%). Most SCIs were incurred in motor vehicle accidents (*n* = 14, 38.9%) and paraplegia was the most common type of injury (*n* = 21, 58%). Most of the participants had complete injuries (*n* = 20, 56%), and the most common NLI was in the thoraco-lumbar region (*n* = 21, 58%). Twenty-six participants (72%) were readmitted to hospital after rehabilitation (Table 1). Most of the participants did not smoke (*n* = 33, 92%) and did not consume alcohol (67%, *n* = 24). There were 21 participants (58%) with paraplegia and 15 (42%) with tetraplegia (Table 1). Figure 1 illustrates that participants with L1–L5 NLI did not meet the expected SCIM III scores (65% vs. 72%) as stipulated by Aidinoff et al. (2011).

**TABLE 1:** Frequency distribution of demographic variables of participants (*n* = 36).

Characteristics	Frequency	Percentage
**Age**				
18–29	5	14
30–39	8	22
40–49	10	28
50–59	7	19
≥60	6	17
**Gender**	
Male	24	67
Female	12	33
**Marital status**			
Single	18	50
Married	13	36
Widowed	2	6
Divorced	3	8
**Qualification**				
None	9	25
Certificate	5	14
Diploma	12	33
Degree	10	28
**Employment status**	
Unemployed	14	39
Employed	22	61
**Cause of injury**	
Traumatic	28	78
Non-traumatic	8	22
**Type of SCI**	
Paraplegia	21	58
Tetraplegia	15	42
**Completeness of injury**	
Complete	20	56
Incomplete	16	44
**Level of injury**					
C1–C4	2	6
C5–T1	13	36
T2–T6	7	19
T7–T12	7	19
L1–L5	7	19
S1–S5	0	0
**Readmission status**	
Readmitted	26	72
Not readmitted	10	28
**Number of readmissions**				
Once	7	19
Twice	3	8
Thrice	5	14
Four times	1	3
≥ five times	10	28

### Health behaviour and participation in leisure-time physical activity

**FIGURE 2:**
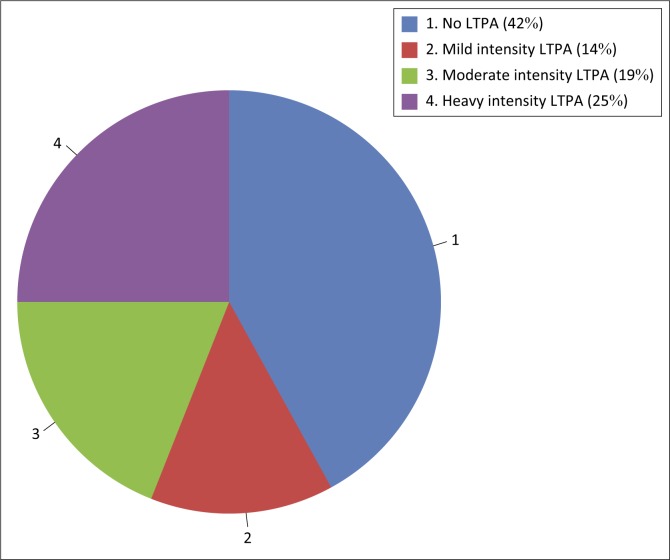
Participation in leisure-time physical activity by people with spinal cord injury using the leisure-time physical activity questionnaire for people with spinal cord injury.

Most participants avoided smoking (*n* = 32, 89%) and always attempted to reduce their risk of heart disease (*n* = 16, 44%). Most participants paid attention to their body position when sleeping (*n* = 23, 64%) and while sitting in the wheelchair (*n* = 16, 44%). Nineteen participants (53%) always performed range of motion (ROM) exercises and 19% (*n* = 7) of participants never performed muscle strengthening exercises. Despite 39% of participants (*n* = 14) always performing some type of pressure relief, 17% (*n* = 6) never inspected their skin for breakdown. Most of the participants were always careful while handling hot liquids (*n* = 35, 97%) as shown in Table 2. The majority of PWSCI (42%) in this study did not participate in any LTPA as shown in Figure 2.

**TABLE 2:** Descriptive statistics of the Spinal Cord Injury Lifestyle Scale (*n* = 36).

SCILS items	Mean	SD
**Cardiovascular**				
I avoid smoking	3.64	1.13
I limit the amount of fat and cholesterol in my diet	2.33	1.72
I am aware of and try to reduce my risk for heart disease	2.58	1.56
I monitor my blood pressure on a regular basis	1.78	1.33
Mean Cardiovascular total	10.33	5.74
**Genitourinary**				
I use an intermittent catheterisation programme and stick to the recommended schedule	1.22	1.87
I change my catheters as often as I have been directed to	2.97	1.75
I have episodes of bladder incontinence	3.00	1.35
I use a rectal suppository as part of my regular bowel programme	2.53	1.78
Mean Genitourinary total	9.72	6.75
**Neuromusculoskeletal**								
I do range of motion exercises daily to keep my joints flexible	3.03	1.28
I do exercises that enhance my muscle strength at least three times a week	2.83	1.38
My muscle strengthening exercises are monitored by a therapist at least once a year	1.89	1.53
I allow my shoulder joints to rest when I am having pain from overusing them	2.83	1.40
I do activities that put weight on the bones in my legs	1.97	1.96
I pay attention to the position my body is in when I am in my wheelchair	2.94	1.19
I pay attention to the position my body is in when I am sleeping	3.19	1.28
If I noticed the beginning of a contracture, I would know exactly what to do	3.22	1.33
Mean Neuromusculoskeletal total	21.92	11.35
**Skin**							
I check my skin to look for any areas of redness or breakdown	2.86	1.50
I do some type of pressure relief every 30 minutes any time I am in my chair or driving	2.89	1.12
I am careful not to bump my legs, feet, or buttocks when doing transfers	3.69	0.82
I wear something on my feet when I am out of bed	3.81	0.53
I am careful when handling hot liquids by not carrying them in my lap	3.97	0.17
I am aware of the condition of my wheelchair cushion	2.86	1.61
I am aware of the condition and repair needs of my wheelchair	2.86	1.61
Mean Skin total	22.94	7.36
**Psychosocial**			
I am able to get around my house	3.42	0.77
I am with or talk to other people at least once a day	3.94	0.23
Mean Psychosocial total	7.36	1.00
Mean SCILS total	72.27	32.2

SCILS, Spinal Cord Injury Lifestyle Scale; SD, standard deviation.

### Secondary health conditions

The most common SHCs, causing significant problems for participants, were chronic pain (*n* = 17, 47%), muscle spasms (*n* = 13, 36%) and joint and muscle pain (*n* = 8, 22%). Only 14% (*n* = 5) of participants reported PUs and bowel dysfunction as significant problems. Burns were the least common SHC with only one participant reporting it as a mild problem. The majority of the participants (*n* = 25, 69.4%) reported that PUs were not a problem in the last 3 months, while five participants (13.9%) reported PUs as a significant problem (Table 3).

**TABLE 3:** Secondary health conditions experienced by people with spinal cord injury as well as the descriptive statistics of the Spinal Cord Injury Secondary Conditions Scale (*n* = 36).

Secondary health condition	Significant problem (%)*	Moderate problem (%)	Mild problem (%)	Not a problem (%)	Mean	SD
Chronic pain	17 (47.2)	3 (8.3)	4 (11.1)	12 (33.3)	1.69	1.37
Muscle spasms (spasticity)	13 (36.1)	4 (11.1)	7 (19.4)	12 (33.3)	1.50	1.30
Joint and muscle pain	8 (22.2)	5 (13.9)	5 (13.9)	18 (50.0)	1.08	1.25
Bowel dysfunction	5 (13.9)	4 (11.1)	12 (33.3)	15 (41.7)	0.97	1.06
Circulatory problems	5 (13.9)	3 (8.3)	12 (33.3)	16 (44.4)	0.92	1.05
Pressure ulcer(s)	5 (13.9)	2 (5.6)	4 (11.1)	25 (69.4)	0.64	1.10
Autonomic dysreflexia	5 (13.9)	1 (2.8)	9 (25.0)	21 (58.3)	0.72	1.06
Sexual dysfunction	5 (13.9)	1 (2.8)	2 (5.6)	28 (77.8)	0.53	1.08
Contractures	4 (11.1)	4 (11.1)	4 (11.1)	24 (66.7)	0.67	1.07
Bladder dysfunction	3 (8.3)	3 (8.3)	10 (27.8)	20 (55.6)	0.69	0.95
Postural hypotension	2 (5.6)	4 (11.1)	9 (25.0)	21 (58.3)	0.64	0.90
Urinary tract infections	2 (5.6)	2 (5.6)	3 (8.3)	29 (80.6)	0.36	0.83
Heterotopic bone ossification	1 (2.8)	0 (0.0)	2 (5.6)	33 (91.7)	0.14	0.54
Diabetes mellitus	1 (2.8)	0 (0.0)	0 (0.0)	35 (97.2)	0.08	0.50
Respiratory problems	0 (0.0)	2 (5.6)	5 (13.9)	29 (80.6)	0.25	0.55
Burns	0 (0.0)	0 (0.0)	1 (2.8)	35 (97.2)	0.03	0.17

**Mean total**	**5 (13.2)**	**2 (6.6)**	**6 (15.4)**	**23 (64.7)**	**10.91**	**14.78**

SD, standard deviation.

* , Ranked by most to least participants reporting significant problems.

### Quality of life and social support

Most participants (*n* = 21, 58%) rated their QOL as good, while 33% (*n* = 12) were neither satisfied nor dissatisfied with their health. Only two participants (6%) did not enjoy their lives at all as compared to eight participants (22%) who really enjoyed their lives. Eleven participants (31%) found their lives to be very meaningful, while 10 participants (28%) found their lives to be moderately meaningful. Thirteen participants (36%) were satisfied with their ability to work as compared to the eight participants (22%) that were dissatisfied. Most participants (*n* = 19, 53%) were satisfied with themselves and their personal relationships, and 14 participants (39%) were satisfied with their sex life. Extreme physical pain limited 47% of participants (*n* = 17) in their daily activities, with nine participants (25%) needing medical treatment to function in their daily life. Most participants reported negative feelings, such as depression (*n* = 34, 94%). Nine participants (25%) reported that they always had negative feelings, 13 (36%) reported the feelings very often, seven (19%) quite often and five (14%) reported the feelings as occurring occasionally (Table 4). Most participants (*n* = 19, 53%) reported that they received help ‘very often’ in special circumstances. However, 28% of participants were rarely invited to a party or dinner.

**TABLE 4:** Descriptive statistics of the World Health Organization Quality of Life Assessment-BREF scores (*n* = 36)

WHOQOL-BREF items	Mean	SD
**Overall Quality of Life and General Health**		
How would you rate your quality of life?	3.67	1.12
How satisfied are you with your health?	3.19	1.19
Mean Overall Quality of Life and General Health total	6.86	2.11
**Domain 1– Physical Health**							
To what extent do you feel that physical pain prevents you from doing what you need to do?	3.97	1.23
How much do you need any medical treatment to function in your daily life?	3.03	1.58
Do you have enough energy for everyday life?	3.58	1.00
How well are you able to get around?	3.50	1.06
How satisfied are you with your sleep?	3.61	0.93
How satisfied are you with your ability to perform your daily living activities?	3.47	1.11
How satisfied are you with your capacity for work?	2.97	1.18
Mean Physical Health total	24.14	4.87
**Domain 2 – Psychological**						
How much do you enjoy life?	3.42	1.16
To what extent do you feel your life to be meaningful?	3.61	1.10
How well are you able to concentrate?	3.89	1.00
Are you able to accept your bodily appearance?	3.86	1.18
How satisfied are you with yourself?	3.86	1.00
How often do you have negative feelings such as blue mood, despair, anxiety, depression?	3.61	1.18
Mean Psychological total	22.25	4.99
**Domain 3 – Social Relationships**			
How satisfied are you with your personal relationships?	4.14	0.68
How satisfied are you with your sex life?	3.17	1.18
How satisfied are with the support you get from your friends?	4.08	0.87
Mean Social Relationships total	11.39	2.018
**Domain 4 – Environment**								
How safe do you feel in your daily life?	3.33	1.01
How healthy is your physical environment?	3.64	0.68
Have you enough money to meet your needs?	2.94	1.09
How available to you is the information that you need in your daily-to-day life?	3.97	1.11
To what extent do you have the opportunity for leisure activities?	3.25	1.16
How satisfied are you with the condition of your living place?	3.89	0.92
How satisfied are you with your access to health services?	3.97	1.11
How satisfied are you with your transport?	3.56	1.34
Mean Environment total	28.56	5.39

**Mean WHOQOL-BREF total**	**64.78**	**16.53**

WHOQOL-BREF, World Health Organization Quality of Life Assessment; SD, standard deviation.

### Associations between health behaviour, secondary health conditions and quality of life

#### Health behaviour

Overall, health behaviour had a moderate correlation with QOL (*r* = 0.457, *p* < 0.01) and social support (*r* = 0.425, *p* < 0.01). Specific genitourinary health behaviour had a negative and moderate correlation with the PWSCIs’ overall independence (SCIM III score) (*r* = -0.468, *p* < 0.01). People with SCI who never rest their shoulders when painful reported the presence of joint and muscle pain as a significant problem, and those who rested their shoulders reported the opposite, suggesting that the two variables are associated. This association is confirmed by a chi-square test of independence, [*x*^2^ (12) = 108.0, *p* < 0.001].

#### Presence of secondary health conditions

The overall presence of SHCs showed moderate and negative correlations with the neuromusculoskeletal health behaviour (NmsHB) (*r* = -0.426, *p* < 0.01). The presence of SHCs only showed low and negative correlation with respiratory and sphincter independence and functional independence (*r* = -0.388, *p* < 0.05 and *r* = -0.343, *p* < 0.05 respectively). People with SCI who did not report burns as a SHC (97%) reported to being careful when handling hot liquids and the chi-square test confirmed the association [*x^2^* (1) = 36.00, *p* < 0.001]. Sixty-seven per cent of PWSCI who did not report contractures as a SHC reported knowing exactly what to do when noticing the beginning of a contracture and the chi-square test confirmed the association [*x^2^* (9) = 108.00, *p* < 0.001]. People with SCI who did not report chronic pain as a problem were very satisfied with their health, while those who reported chronic pain as a significant problem were very dissatisfied with their health. This finding suggested an association between the two variables, which was confirmed by a chi-square test [*x*^2^ (12) = 23.856, *p* < 0.05]. Furthermore, of the 47% (*n* = 17) who reported chronic pain as a significant problem, 88% (*n* = 15) reported needing medical treatment to function in their daily life [*x*^2^ (12) = 22.503, *p* < 0.05]. Of the 50% of PWSCI who reported joint and muscle pain as a problem (Table 3), 33% (*n* = 6) reported the pain as a significant problem and they all felt that the physical pain prevented them from doing what they need to do (physical QOL) [*x*^2^ (12) = 23.702, *p* < 0.05]. People with SCI who reported bladder dysfunction (such as incontinence) reported feeling depressed very often [*x*^2^ (12) = 28,60, *p* < 0.01].

#### Quality of life and social support

Overall, QOL had a moderately high correlation with social support (*r* = 0.671, *p* < 0.001) and a moderate correlation with skin health behaviour (SHB) (*r* = 0.517, *p* < 0.01). Psychological health and social relationships QOL also showed moderate correlations with SHB (*r* = 0.421, *p* < 0.01 and *r* = 0.446, *p* < 0.01, respectively). People with SCI who were satisfied with their personal relationships reported to be aware and always attempted to reduce their risk for heart disease as compared to those who were dissatisfied with their personal relationships [*x*^2^ (8) = 17.159 *p* < 0.05]. Those who were dissatisfied with the support they received from their friends did not adequately perform the ROM exercises, and those who were very satisfied with the support received from their friends almost always performed ROM exercises [*x*^2^ (12) = 28,190 *p* < 0.01].

## Discussion

### Outline of the results

The purpose of this study was to investigate the associations among health behaviour, SHCs and QOL in PWSCI. Participation in specific health-benefiting behaviour was found to be associated with the presence of SHCs, QOL and social support from family and friends. Health behaviour, the presence of SHCs and QOL varies with each individual after SCI owing to the different impairments that present after injury. The impairments (which are deviations from the normal functioning of body structures and functions) may be temporary or permanent; progressive or regressive; mild or severe and may be intermittent or continuous (WHO 2001). Because of the impairments being different in different individuals, participation in health behaviour, the presence of SHCs and the reporting of QOL will differ accordingly. It is therefore understandable that specific impairments following a SCI predispose PWSCI to developing specific SHCs.

Persistent and severe pain is common after SCI (Widerström-Noga et al. 2016) and we found neuropathic pain to be the most common SHC in our study. The prevalence of pain has been reported in 11% – 94% of PWSCI, and pain is found to be associated with poorer psychological functioning and reduced QOL (Andressen et al. 2016). Our findings are therefore not surprising, as PWSCI in our study who reported chronic pain as a significant problem were very dissatisfied with their health. The problem of pain following SCI has been rated as one of the most difficult problems to manage, as it directly contributes to disability and reducing of QOL and life satisfaction (Siddall 2009). In this study, half of the PWSCI reported nociceptive pain in the form of joint and muscle pain in the shoulders. Our findings are in line with literature, as an estimated 67% of PWSCI suffer from musculoskeletal shoulder pain, with prevalence rates ranging from 30% to 78% (Eriks-Hoogland et al. 2014; Morrow et al. 2010; Rafiullah, Shah & Mazhar 2017). The lifelong dependency of PWSCI on wheelchairs for activities of daily living (ADLs) and mobility causes repetitive loading of the shoulder joint which can increase the stress on the shoulder joint, thereby causing shoulder pain (Cardenas & Felix 2009). People with SCI may feel that the presence of pain prevents them from doing what they need to do, but because of dependency on their upper limbs they may continue to load their shoulders despite the pain. It was therefore not surprising that we also found that PWSCI with joint and muscle pain did not rest their shoulders. This behaviour is understandable considering that PWSCI depend on their upper limbs for daily activities, transfers and propelling their manual wheelchairs (Eriks-Hoogland et al. 2014). The increased use of the upper extremities may exacerbate shoulder pain, possibly leading to joint degeneration, placing PWSCI at risk of surgical interventions. Daily and constant use of medication to function (as found in this study) increases the feeling of dependency after SCI and may lead to an increased risk of depression and decreased health satisfaction. Although this study did not find any association between pain and depression, there is increasing evidence emerging that depression often coexists with pain and higher proportions of PWSCI have more depressed mood than able-bodied people (Ataoğlu et al. 2013). This study, however, did find an association between bladder incontinence and depression, which is supported by Liu et al. (2010), who also found that incontinence has a negative influence on the emotions of PWSCI.

We did not expect the low correlations between overall presence of SHCs and the non-modifiable injury characteristics of PWSCI (such as level of injury), the poor respiratory and sphincter management as well as functional independence scores according to the SCIM III. Instead, the overall presence of SHCs was found to be associated with poor engagement in NmsHB. This suggests that most of the SHCs after SCI are not only reliant on the type and severity of the SCI but also on the health behaviour of PWSCI. This then means that SHCs can be significantly delayed or prevented by adopting good health behaviour, as supported by Pruitt et al. (1998). Our findings are therefore in line with literature, as Bloemen-Vrencken et al. (2007) previously suggested that PWSCI might experience fewer SHCs if they performed good health behaviours. Good health behaviours can significantly delay SHCs, such as PUs, shoulder joint degeneration and pain, and UTIs, among others (Pruitt et al. 1998). Contrary to expectations, PUs were not a significant problem, ranking fourth in the top five SHCs in our findings. Only five participants (13%) reported PUs as a problem. Pressure ulcers are one of the most common SHCs in the SCI population and numerous authors have confirmed this in studies of prevalence, risk factors and impact on QOL (Marin, Nixon & Gorecki 2013; Mathew et al. 2013; Scheel-Sailor et al. 2013). The low prevalence of PUs in our study may be because of small sample size (*n* = 36) or all PWSCI being discharged from a private rehabilitation centre. The low prevalence of PUs in this study is also in line with the good skin health behaviour reported by the PWSCI. Most PWSCI said they ‘almost always’ inspect their skin for redness or skin breakdown (*n* = 19, 53%), are careful not to bump their buttocks during transfers (*n* = 30, 83%) and are aware of the condition of their wheelchair and cushion (*n* = 22, 61%). We found that the least common SHCs in this study were associated with good health behaviours. Following good health behaviour in terms of burns and contracture prevention yielded low presence of these specific SHCs. This may suggest knowledge on what to do (and what not to do) may assist PWSCI in performing good health-benefiting behaviour.

This study did not find any association between participating in LTPA and QOL, which is in contrast with findings by Tomasone et al. (2013). They found overall LTPA to be positively associated with physical, psychological and social QOL. Almost half of PWSCI in our study did not engage in LTPA, similar to findings by Martin Ginis et al. (2010) and only 31% (*n* = 11) occasionally performed strength exercises. Our results are not surprising, as PWSCI have been found to comprise one of the most inactive population sectors in society (Arbour-Nicitopoulos, Martin Ginis & Latimer 2009). This may suggest that PWSCI may perceive their physical limitations as barriers to LTPA, and would rather not attempt LTPA despite the benefits of participating in LTPA, which include increased strength and flexibility. For PWSCI, the achievement of physical goals may lead to a more positive subjective QOL and greater satisfaction with physical ability. Although the majority of PWSCI in our study did not perform strengthening exercises and LTPA, 53% (*n* = 19) always performed ROM exercises. We also found that PWSCI who were dissatisfied with their friends’ support did not adequately perform ROM exercises. This was an interesting find, as it suggests that motivation to perform exercises necessary for joint health is associated with external motivation from people who do not reside with PWSCI. Suzuki et al. (2007) found that the presence of SHCs is not only related to demographic and personal factors but also to the availability of emotional support. This then suggests that less social isolation and more interpersonal support would be anticipated to lead to fewer SHCs owing to social supports’ positive correlation with QOL. Our findings are thus in line with Suzuki et al. (2007), as we found that PWSCI with good social support system try to reduce their risk of heart disease. Social support is usually associated with the feelings of being cared for, or protected. Having social support for PWSCI means increased opportunity for social activities and connection with friends and family. It is then not surprising that this study found that PWSCI with increased social support also reported higher QOL. This suggests that improved QOL is not only dependent on the absence of SHCs through improved health behaviour but also on improved social relationships through social support. As such, interpersonal support will then directly help people with SHCs to effectively manage circumstances related to their SCI (Suzuki et al. 2007).

### Practical implications

This study has increased the amount of literature in South Africa on the problem of SHCs in PWSCI. This study has further identified the SHCs most experienced by PWSCI, thus enabling rehabilitation health professionals to focus on educating PWSCI at risk of specific SHCs on different SHCs prevention measures. The specific SHCs identified will assist rehabilitation health professionals to focus on addressing the specific factors that increase the risk of developing SHCs as well as introduce ways to maintain good social relationships with family and friends of PWSCI. This study determined which aspects of health behaviour influence the development of different SHCs as well as the impact SHCs have on QOL in PWSCI residing in South Africa, thus bridging the literature gap. The information gained will be useful in developing programmes that may be implemented to prevent the development of SHCs and in so doing improve the QOL of PWSCI.

## Limitations of the study

This study was hindered by the small sample size, where only 36 participants consented instead of the anticipated 60 participants. The study also focussed on PWSCI that were discharged from a private rehabilitation institution, who resided in the CoT metropolitan area. There is a possibility that PWSCI from a different metropolitan area in another province may present with different results. Furthermore, the possibility exists that PWSCI from a government rehabilitation institution may present with different health behaviours, SHCs and QOL owing to different reasons. Despite the abovementioned limitations, this study adds to the body of literature from South Africa on the problem of SHCs in PWSCI.

## Recommendations

This study has revealed that there is a trend among health behaviour, SHCs and QOL in PWSCI. This study can be broadened to include PWSCI in other metropolitan areas, at both government and private rehabilitation institutions. Further research is required to determine the factors that influence health behaviour as well as ways to improve the QOL of PWSCI through social relationships.

## Conclusion

Engagement in health-benefiting behaviours was found to be associated with SHC, QOL and social support. Participating in health-benefiting behaviour (with the help of a good social support system) can reduce the development of SHCs and subsequently increase QOL in PWSCI. Further research is required to determine the factors that influence health behaviour in order to develop strategies to increase health-benefiting behaviours. Rehabilitation health professionals must maintain focus on minimising the development of SHCs by providing specific education on good health-benefiting behaviour, as well as introducing social education to increase support from friends and family.
